# Assessing patients' experience of integrated care: a survey of patient views in the North West London Integrated Care Pilot

**DOI:** 10.5334/ijic.1453

**Published:** 2014-06-09

**Authors:** Nikolaos Mastellos, Laura Gunn, Matthew Harris, Azeem Majeed, Josip Car, Yannis Pappas

**Affiliations:** Department of Primary Care & Public Health, School of Public Health, Imperial College London, UK; Department of Integrative Health Science, Stetson University, FL, USA; Public Health, Department of Primary Care & Public Health, Imperial College London, UK; Primary Care, Department of Primary Care & Public Health, Imperial College London, UK; Primary Care, Department of Primary Care & Public Health, Imperial College London, UK; Health Services Research, Institute for Health Research, University of Bedfordshire, UK

**Keywords:** integrated care, patient experience, care planning, quality of care

## Abstract

**Introduction:**

Despite the importance of continuity of care and patient engagement, few studies have captured patients' views on integrated care. This study assesses patient experience in the Integrated Care Pilot in North West London with the aim to help clinicians and policymakers understand patients' acceptability of integrated care and design future initiatives.

**Methods:**

A survey was developed, validated and distributed to 2029 randomly selected practice patients identified as having a care plan.

**Results:**

A total of 405 questionnaires were included for analysis. Respondents identified a number of benefits associated with the pilot, including increased patient involvement in decision-making, improved patient–provider relationship, better organisation and access to care, and enhanced inter-professional communication. However, only 22.4% were aware of having a care plan, and of these only 37.9% had a copy of the care plan. Knowledge of care plans was significantly associated with a more positive experience.

**Conclusions:**

This study reinforces the view that integrated care can improve quality of care and patient experience. However, care planning was a complex and technically challenging process that occurred more slowly than planned with wide variation in quality and time of recruitment to the pilot, making it difficult to assess the sustainability of benefits.

## Introduction

The number of people with long-term conditions in England is increasing with over 15 million people having one or more chronic illnesses [1]. The coordination of care for those patients is extremely complex as it involves multiple health professionals in a variety of settings. The increasing involvement of various providers in care delivery requires collaborative working to ensure smooth movement of patients across services and settings. However, health care systems often fail to achieve continuity, resulting in poor quality of care, service duplication, extra costs and compromised patient safety [2]. Integrated care has the potential to improve continuity of care and enhance quality, safety, cost-effectiveness and access to services [3–8]. Central to any integration initiative is to ensure that services are coordinated around the needs of patients, carers and service users to provide a seamless care experience [9–11]. As the emphasis of integrated care is on improving quality of care and patient experience, there is a pressing need for research that captures and discusses the perceptions of patients in relation to numerous aspects of care.

Patient experience can encompass various dimensions of continuity of care. Seamless flow of information across care levels [12]; effective communication between providers, patients and services [13, 14]; comprehensiveness and timely access to health care services [15]; a designated professional responsible for coordinating a patient's journey across care boundaries [16]; and flexibility in adjusting to the changing needs of patients [17] are also all considered important components of any integrated care system. Other aspects of care that closely correlate with positive patient experience include patient involvement in decisions about the management of their condition, willingness to listen to patients and explain results from clinical tests and knowledge of their medical history [18, 19].

Previous research has measured several aspects of integrated care, including access to health care services, effect on clinical outcomes and cost-effectiveness [20–22]. However, despite the direct impact of good or poor care on patients' health status, little research has been done to measure integrated care from their perspective, limiting our understanding of what it looks like for patients. Given the current focus on placing patients in the centre of health care interventions, there is an increasing need for research that captures patients' experiences to ensure that transformation meets their needs. The focus of this study is on patient experience of the Integrated Care Pilot in North West London. The primary aim of the study was to capture and understand the perceptions and experiences of patients who had joined the pilot between June 2011 and May 2012 and who potentially had a care plan with a primary care provider. We also aimed to develop a frame of knowledge to facilitate decision-making at policy level and inform future initiatives. Results from the one year pilot, including changes in care processes and health outcomes, are reported elsewhere [23]. Here, we present additional findings resulting from in-depth analyses of patients' responses to provide a detailed and accurate picture of how patients experienced integrated care and enable useful conclusions to be drawn about the impact of integrated care on various aspects of patient experience.

## The Integrated Care Pilot in North West London

North West London is a geographical region with the fastest growing population in the UK, characterised by high prevalence of chronic conditions (especially amongst ethnic minority groups who account for 35% of the population), with nearly one in six people having at least one of the following conditions: diabetes, asthma, coronary heart disease and chronic obstructive pulmonary disease [24, 25]. In mid-2010, clinicians, managers, patients and local authority representatives identified as a strategic priority to move towards integrated care. The Integrated Care Pilot was launched in June 2011 encompassing about 38,000 patients (i.e. approximately 8700 patients with diabetes over 75 years of age, nearly 22,800 non-diabetic elderly patients and around 6500 people with diabetes under 75) from approximately 100 primary care practices in North West London covering a total population of about 550,000 people [26]. It was considered an example of “vertical” integration of a “virtual” network of organisations that operate across care levels [27]. The vision was to significantly improve the quality and experience of care for patients with diabetes and the elderly, create access to better and more coordinated care outside of hospital, reduce unnecessary admissions and enable effective inter-professional communication and collaboration across care levels [24]. The pilot is described in more detail elsewhere [26]. Briefly, participating practices were offered incentives to develop specific, bespoke care plans for registered patients with diabetes and/or that were over 75 years of age. By developing care plans together with the patient, general practitioners were able to identify patients at risk of hospitalisation and develop strategies in multi-disciplinary case discussion meetings to coordinate care across services in primary and secondary care, and in the community, thereby reducing hospital admissions.

## Methods

The overall mixed-methods evaluation methodology for the whole project has been described [28], as have results from other aspects of the pilot evaluation [23, 26, 29, 30]. In this paper, we focus on one aspect of the evaluation: patient experience. A cross-sectional survey design was adopted to assess patients' experience with the pilot. A structured, five-point Likert-scale questionnaire was developed in three stages. First, a literature review was undertaken to identify common themes of patient experience with integrated care and care planning. Patient–provider relationship, patient involvement in decision-making, communication, coordination, quality of care and access to services were the most important aspects identified in the literature. Second, based on these findings, a questionnaire was designed through consensus amongst a multidisciplinary team of researchers at Imperial College, consisting of sociologists, psychologists and clinicians, and underwent internal validation through face validity assessment in a series of weekly meetings over a 6-month period. The questionnaire initially consisted of 22 items, but 3 items were removed as they were found to either overlap with other items (e.g. “I feel my care has been better”, “I feel my care has overall improved”) or cause confusion (e.g. “my care has felt more ‘joined up’”). As a result, a 19-item questionnaire measuring patient experience with integrated care (ten items) and care planning (nine items) was developed. The survey was piloted with a convenience sample of seven general practice patients who had consented to take part in an interview assessing patient experience with the pilot. Participants were asked to complete the survey and describe whether it reflected their experiences accurately and unambiguously, as well as whether there was anything missing. Their feedback enabled us to reword certain survey items so that the questions become clearer and simpler (e.g. “I know more about who is involved in my care” was changed to “I know more about which health professionals are involved in my care”).

The eligibility criteria included patients with type-2 diabetes and/or elderly persons (over 65) who had consented to participate in the pilot and were identified by the IT system (i.e. a tool designed to extract and use data from general practices, acute care trusts, community services and mental health care services, with the aim to facilitate the care planning process by making care plans accessible to a range of providers involved in a patient's care) as having a care plan. Care plans were created and agreed upon by the patient and the professionals participating in their care within approximately a month after a person consented to have their information shared. A search on the integrated care pilot's database generated 18,484 patients who had consented to join the pilot (as per 31st May 2012). Of those, 13,322 were excluded due to incorrect entry of their contact details, which would have likely affected receipt of the questionnaire, resulting in a total of 5162 eligible patients from whom to sample. Due to budget constraints, a convenience sample of 2029 participants, out of the identified 5162 eligible patients with full contact details, was selected using a random number generator on Excel to control for selection bias to the possible extent. Paper-based, self-completed questionnaires were distributed to the selected 2029 patients from primary care practices in North West London between June and July 2012.

Questionnaires were analysed using the Statistical Package for Social Sciences (v19). Descriptive statistics were used to identify the relative proportion of patients who were satisfied or dissatisfied with different aspects of the pilot as per item on the questionnaire. An available case analysis was used for each variable to handle missing data. Separate analyses were performed on subsets of patients who were aware of having a care plan to assess their satisfaction with their involvement in the development of their care plan. Data were categorised into negative (i.e. strongly disagree, disagree somewhat), positive (i.e. strongly agree, agree somewhat) and neutral (i.e. neither agree nor disagree, I do not know) responses in order to distinguish more clearly the direction of responses. *χ*^2^-tests were used to explore the relationship between time-in-pilot and patient experience, as well as to determine the association between patients who were aware of having a care plan and satisfaction with different aspects of the pilot. Odd ratios were computed to describe the strength of association amongst the study variables by care plan status and time-in-pilot. Ethical approval for this study was granted by the National Health Service National Research Ethics Service for City and East London (ref. 11/LO/1918).

## Results

A total of 472 of the 2029 surveys were returned yielding a response rate of 23%. However, 67 questionnaires included only demographic data and were excluded, restricting the analysis to the remaining 405 patients (overall response rate 20%). The characteristics of the respondents are broadly comparable, as shown in Table 1. Most respondents (53.5%, *n* = 207) could not recall when they had consented to join the pilot and only a minority (15.5%, *n* = 60) had been recruited in the first half of the one-year pilot. Of the 405 respondents, only 22.4% (*n* = 88) were aware that they had a care plan; and of these, only 37.9% (*n* = 33) replied that they had a copy of their care plan.

The dominant perception was that the pilot resulted in a feeling of involvement in decisions about their care with nearly seven in ten respondents (68.9%, *n* = 262) sharing this view amongst all participants (*n* = 405). The data also show that the intervention had a perceived positive impact on other aspects of the patient–provider relationship. Specifically, 61.7% (*n* = 230) of respondents felt that their relationship with their general practitioner had improved as a result of the pilot. Moreover, the pilot improved their knowledge about which health professionals are involved in their care (50.7%, *n* = 185), increased their expectations during patient–provider encounters (53.5%, *n* = 192) and resulted in health care staff asking fewer questions about their medical history (54.4%, *n* = 203). Most patients also reported that they had easier access to health services (57.6%, *n* = 212), though not quite half (46.1%, *n* = 166) needed to do less work to organise their care (e.g. chasing up people to organise appointments) as a result of the pilot. Despite a general consensus amongst respondents on the positive impact of the pilot, only 18.3% (*n* = 66) said they experienced changes at the point of care provision (Table 2).

Knowledge of having a care plan was highly significantly associated with a more positive patient experience across all domains measured in this study (Table 3). Odds ratios (95% CI) and *p* values (*χ*^2^ tests) all provide strong evidence for these highly significant associations yielding a more positive patient experience amongst respondents who knew they were on a care plan compared to those who were not aware of having one despite being part of the pilot. Most notably, patients who were aware that they had a care plan were nearly 18 times (OR 17.57 95%CI 2.37–130.26; *p* = 0.0003) more likely to feel more involved in decision-making compared to patients without knowledge of having a care plan. In addition, patients who knew they had a care plan demonstrated significantly greater awareness of who is involved in their care (OR 7.44 95%CI 2.58–21.45; *p* < 0.0001) and what to expect from those providing care (OR 8.12 95%CI 2.42–27.22; *p* = 0.002). Significant differences amongst care plan groups were reported in all other aspects of patient experience, including patient–provider relationship (OR 4.0 95%CI 1.37–11.65; *p* = 0.0117), inter-professional communication (OR 5.04 95%CI 1.91–13.33; *p* = 0.0008), care coordination (OR 4.28 95%CI 1.92–9.57; *p* = 0.0003), access to health services (OR 5.36 95%CI 2.05–14.0; *p* = 0.0003), patient–provider communication (OR 4.77 95%CI 1.95–11.66, *p* = 0.0004) and quality of care (OR 4.4 95%CI 1.30–14.93; *p* = 0.0185).

Time since recruitment to the pilot was generally associated with more positive patient experiences. However, the relationship between time-in-pilot and patient experience did not reach significance, with *p* values for *χ*^2^-tests of association ranging from 0.116 to 0.953 (Table 4).

Patients who indicated having a care plan welcomed the new way of care planning (Table 5). Most importantly, they said they understood how their care plans work (78.8%) and felt involved in planning their care the way they wanted it to be (65.1%). There was also a general agreement in relation to one fundamental principle of integrated care: inter-professional communication. The vast majority of respondents with knowledge of their care plan status (94.1%) replied that all health care professionals involved in the management of their care should share information with one another. However, one in three respondents (36.4%) were not involved in creating their care plan, suggesting that the pilot may be missing opportunities for maximising the potential for self-management.

## Discussion

Our results show positive patient experience with the Integrated Care Pilot. However, they also reveal deficiencies in the care planning process as most participants were not aware of having a care plan with their local primary care practice by the time of the study. Patient satisfaction was significantly greater amongst respondents who indicated that they had a care plan. The variations in care plan awareness did not allow us to fully explore the impact of the pilot on the care planning process, as the analysis (Table 5) included only the 22.4% of respondents who said they had a care plan. In addition, most patients joined the pilot in the second half of the one-year programme (i.e. approximately 1500 patients in November 2011 compared to 18,500 in May 2012). This was also reflected in the survey with double of respondents replying that they were part of the pilot for less than 6 months (Table 1). Despite being part of the pilot for a short time, however, patients identified a number of benefits and opportunities associated with the launch of the pilot. Those include: increased patient involvement in decision-making; improved patient–provider relationship; better organisation and access to care; and enhanced communication amongst health care providers.

Patient involvement should be at the heart of any integrated care intervention. However, a recent study assessing patient experience with integrated care in England revealed that patients often feel less involved in decisions about their care after being part of the pilot for 12 months [31]. These results indicate that amongst patients who were aware of having a care plan, the pilot has been relatively successful in actively engaging patients in the planning of their care for potentially better management of their overall health.

Nonetheless, it is concerning that over three-quarters of the sampled participants were not aware of having a care plan within a year of the launch of the pilot when the IT system identified these patients as having a care plan. This could be because of information management issues with the IT system that have been described elsewhere [23], or inadequate involvement of patients in the care planning process, or because respondents had forgotten they had been given a care plan. Patients may have also misunderstood what is meant by a care plan and, finally, providers may have been wrongly entering that care planning had taken place when it actually had not. Complex incentive structures to encourage care planning may be susceptible to abuse [26]. Future integrated care initiatives may consider alternative ways to improve patient awareness and use of care plans, such as providing patients with both a paper-based and an electronic copy of their care plan, or using an online patient portal where care plans could be stored, viewed and updated by clinicians and, to some extent, patients.

Good communication between health care providers and patients is essential to improve planning and delivery of services [32, 33]. Equally important is to ensure that all parties have a clear understanding of their role and the role of others within the relevant context to enable effective collaboration [34]. The pilot enhanced patients' knowledge about which health care professionals are involved in their care reinforcing the view that perceived role clarity is associated with positive patient experience [14, 34]. Patient–provider relationship breakdowns have been repeatedly reported as a main barrier to the provision of integrated care [13, 14, 19]. Listening to the views of patients and measuring whether the services being delivered meet their needs can help to improve both quality of care and patient experience.

The analysis shows that patients were generally happy with the access to health and social care services, and with needing to do less work to organise their care. Patients seen in integrated care settings are expected to report high levels of satisfaction with access to care, including follow-up hospital and general practice appointments, community services and after-hours services [3]. Our findings support the results of previous studies looking at patient experience with integrated care [31, 35]. This is particularly important in an integrated care context where the overall aim is to enable a seamless move of patients across care levels.

The findings also reveal some impact of the pilot on cross-boundary communication and highlight the patients' support for collaborative working. Effective collaborative working and communication across care levels are critical to the provision of high-quality integrated care [32, 33, 36]. Communication, even perceived communication between providers, is an essential component of any integrated care system. In our study, respondents were more likely to notice changes in communication the longer they were enrolled in the pilot.

Additionally, despite significant differences in how patients who knew they had a care plan and those who did not know experienced integrated care, it is noteworthy that the latter also had an overall positive experience. Most importantly, six out of ten felt involved enough in decisions about their care and one in two responded that the quality of care had improved following the launch of the pilot. These results may reflect the general satisfaction with the quality of care provided in the National Health Service or may insinuate some kind of response bias stemming from the subjective interpretations or expectations of those who participated in the study.

This study has some limitations. The majority of the respondents had been enrolled in the pilot for only a short period of time and had no awareness of their care plan. Considering that integrated care needs time to establish successful structures and deliver the anticipated benefits [3, 35, 37], a one-year pilot may have been too short to notice changes in care delivery, especially for respondents who joined the pilot in the last 6 months or those who had limited contact with the NHS over that period. Integrated care interventions need to be monitored over the long-term to capture their effect on patient experience and quality of care [31]. Another limitation is that, although survey respondents were broadly similar in terms of age and gender, socioeconomic data, such as ethnicity, social status and educational background, were not collected to enable comparison of their characteristics to those of the broader population of interest, potentially introducing selection bias. Recall bias and willingness-to-please may have affected the findings as is often the case with surveys although we offered no financial incentive to complete the survey. We attempted to improve the validity of our survey instrument through multidisciplinary consensus and cognitive interviewing with a pilot sample. Given the relatively low response rate, our sample represents less than 1% of the enrolled patients in the pilot. Provided that no power calculation was conducted, we cannot really estimate whether the sample is powered enough to detect any small differences, although it should be sufficient to give responses to a reasonable degree of precision. The small and skewed sample, due to incorrect entry of contact details into the IT system, may restrict the extrapolation of our findings. However, the results provide a useful indication of how patients experience integrated care and call for future research in the field to confirm or reject our findings. Future studies may follow up patients for a longer period to assess whether integrated care has a sustainable impact upon care provision and explore patient experience in post-survey interviews and focus groups with patients and/or their carers.

## Conclusions

This study examines patient experience with the Integrated Care Pilot in North West London, though some of its results mirror that of studies worldwide. It reinforces and extends the work of other researchers in the field to capture the competences required to deliver the anticipated benefits of integrated care. Despite the current emphasis on improving continuity of care and patient engagement, few studies have measured the impact of integrated care on various aspects of patient experience with quality of care through eliciting the views of patients, limiting our understanding of what integrated care looks like for those receiving it. Our study provides empirical evidence that integrated care has the potential to improve patient experience by increasing patient involvement in decision-making, enhancing the patient–provider relationship, strengthening collaborative working and providing easier access to care. In a more than ever patient-centred health care, integrated care models need to reflect the views of patients to ensure that the services provided fit with their values and needs. Listening to the views of the patient can help policymakers and clinicians develop pathways that meet patients' needs and enable them to provide high-quality integrated care.

## Figures and Tables

**Table 1.**
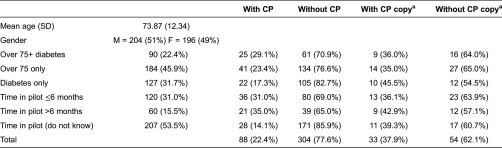
Sample characteristics

^a^Amongst the 22.4% who indicated having a care plan (CP).

**Table 2.**
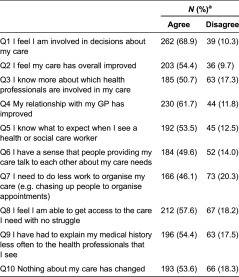
Overall patient experience (*N* = 405)

^a^The remaining percentage (from 100%) for each row and group pertains to neutral responses.

**Table 3.**
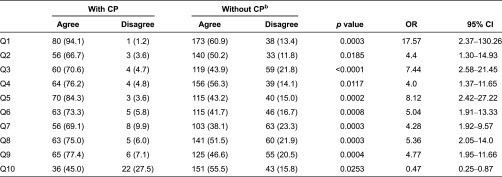
Patient experience according to care plan status, *N* (%)^a^

^a^The remaining percentage (from 100%) for each row and group pertains to neutral responses.

^b^All patients were registered as having a care plan, but the majority were not aware of having one.

**Table 4.**
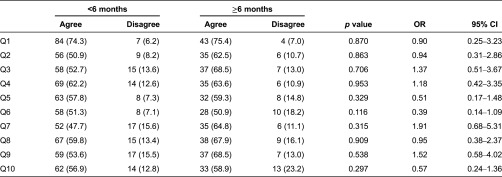
Patient experience per time-in-pilot *N* (%)^a^

^a^The remaining percentage (from 100%) for each row and group pertains to neutral responses.

**Table 5.**
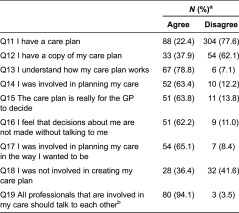
Care planning amongst the 22.4% of patients with CP

^a^The remaining percentage (from 100%) for each row pertains to neutral responses.

^b^Patients without care plans also replied and scored 72.1% and 9.2%, respectively (overall: 77.7% and 7.8%).
